# SOX2 and BCL-2 Expressions in Odontogenic Keratocyst and Ameloblastoma

**DOI:** 10.4317/medoral.23348

**Published:** 2020-01-22

**Authors:** Brunno Santos de Freitas Silva, Lorena Rosa Silva, Kaique Leite de Lima, Amanda Cristina Ferreira dos Santos, Anna Carolina Oliveira, Ana Cláudia Dezzen-Gomide, Aline Carvalho Batista, Fernanda Paula Yamamoto-Silva

**Affiliations:** 1 DDS, MSc, Ph.D. Professor of Oral Pathology, Department of Oral Diagnosis, School of Dentistry, University of Anápolis, Anápolis, GO, Brazil; 2 DDS, MSc. Postgraduate student, School of Dentistry, Federal University of Goiás, Goiânia, GO, Brazil; 3 DDS. Undergratuated student, Department of Stomatologic Sciences, School of Dentistry, Federal University of Goiás, Goiânia, GO, Brazil; 4 DDS. Postgraduate student, School of Dentistry, University of Anápolis, Anápolis, GO, Brazil; 5 DDS, MSc, Ph.D. Professor of Oral Pathology, Department of Stomatologic Sciences, School of Dentistry, Federal University of Goiás, Goiânia, GO, Brazil; 6 DDS, Ph.D. Professor of Oral Radiology, Department of Stomatologic Sciences, School of Dentistry, Federal University of Goiás, Goiânia, GO, Brazil

## Abstract

**Background:**

The purpose of this experimental study was to compare the immunohistochemical expression of SOX2 and BCL-2 in Odontogenic Keratocyst (OKC) and Ameloblastoma (AB) specimens, and to identify a possible correlation in their expression.

**Material and Methods:**

Immunohistochemical analysis was performed to evaluate SOX2 and BCL-2 expression in OKC (n = 20) and AB (n = 20). The immunoexpression was analyzed by a quantitative and qualitative scoring system. The comparison between the immunoexpression of SOX 2 and BCL-2 was assessed by the Mann-Whitney U-test. Spearman’s correlation coefficient evaluated the correlation between SOX2 and BCL-2 expressions.

**Results:**

SOX2 and BCL-2 expression was observed in all specimens of OKC in the full thickness of the epithelium lining. SOX2 immunostaining was higher in OKC, in comparison with AB samples (*P*<0.05). BCL-2 immunostaining between OKC and AB was not statistically significant. There was no significant correlation between SOX2 and BCL-2 in OKC and AB specimens.

**Conclusions:**

SOX2 and BCL-2 expressions in OKC may suggest their relationship with the biological behavior of this lesion, and the higher expression of SOX2 might be an upstream influence on the Hh signaling pathway.

** Key words:**Odontogenic keratocyst; Ameloblastoma; Odontogenic tumor; SOX2; BCL-2.

## Introduction

Odontogenic keratocyst (OKC) is a benign intraosseous lesion of the jaws, and is of odontogenic origin, representing about 11.7% of odontogenic cysts (1). Over the last decades, the nature of odontogenic keratocysts (OKC) has been widely discussed (2). Most of the recent debate has focused on their aggressive behavior and high recurrence rate, as well as the existence of molecular alterations in the OKC. These alterations are regarded as an important factor supporting their classification as a benign cystic neoplasm (3). OKCs present significant changes in PTCH1 and in the Hedgehog (Hh) signaling pathway, and are one of the major molecular alterations found in OKC lesions (4). Although the molecular changes were indeed observed in the OKC specimens, it seems that they are not exclusive to this condition, since similar molecular alterations have also been found in developmental cysts (5). This finding, together with the fact that some OKC lesions respond to marsupialization procedures, puts their neoplastic nature into question (6).

Ameloblastoma (AB) is a benign tumor of the odontogenic epithelium, and also has a recognized aggressive nature, significant growth potential and a high recurrence rate (7). Many studies have attempted to investigate the AB gene expression profile (8-12); and some have suggested that the AB oncogenic transformation is related to abnormalities in multiple genes (11,12). Moreover, several of these investigations have proposed to compare the molecular characteristics of OKC and AB with the intent of understanding their biological behavior and development mechanisms (9,12). Apparently, they seem to be distinct lesions with respect to gene expression, in that AB presents a dental profile, expressing PITX2, MSX2, DLX1, 2, 3, 4, ISL1, TBX1, IRX1, RUNX1 and SOX9, whereas KOCT presents an embryonic oral epithelium profile, expressing SOX2 (9). However, OKC and AB can also present some molecular similarities regarding alterations in Hh (8,12,13), mitogen-activated protein kinase (MAPK)(14) and WNT/B-catenin signaling pathways (10,11,15).

In a previous study, it was found that OKCs present a high level of SOX2 expression, indicating that OKC epithelium could harbor a great number of cells with stem cell characteristics (9). SOX2 is a transcription factor expressed in embryonic and adult stem cells, and exerts an important influence on maintaining pluripotency (16). It has been found to be related to oncogenic signaling pathways, controlling tumor cells by affecting their fate, proliferation and apoptosis (17). In oral primitive epithelium SOX2 is highly expressed, and this expression is also sustained during tooth development (9). SOX2 is not expressed in the epithelial rest cells of Malassez nor in the epithelial cells of Hertwig’s epithelial root sheath, but it is significantly expressed in dental lamina (16), which could probably be the source of OKC development (4). OKC also has an increased expression of BCL-2 (13,18), which is an anti-apoptotic gene with important implications in tumorigenesis and tumor progression (19). There is evidence that there exists some kind of interaction between SOX2 and BCL-2 (20), inasmuch as both genes are related to Hh (13,21), MAPK (22) and WNT/B-catenin (16) signaling pathways. In fact, SOX2 plays a role in activating the Hh pathway, being responsible for sustaining the stem cell phenotype (21), and also has an effect on cell death prevention by influencing BCL-2 levels (20).

Recently, Juuri *et al*. [2013] (16) showed that SOX2 is also expressed in AB and in fragments of dental lamina associated with the developing third molar, which could be a sign that this tissue may give rise to an AB. According to the mentioned study, SOX2 positive odontogenic epithelium may originate AB in this particular anatomic region, because SOX2 positive epithelium apparently preserves the capacity to proliferate and produce dental epithelium.

Since SOX2 and BCL-2 could affect signaling pathways known to exert some sort of influence on OKC and AB, and since these two genes may interact with each other in other tumor models, the study of their expression in OKC and AB seems to be opportune. The understanding of the OKC and AB genetic profile could be beneficial to the elucidation of their pathogenesis and also to the proposal of new treatment modalities (4). Accordingly, the aims of the present study were to compare the immunohistochemical expression of SOX2 and BCL-2 in OKC and AB specimens, and to identify a possible correlation in their expression.

## Material and Methods

- Study design and specimens

This experimental study included an immunohistochemical analysis of SOX2 and BCL-2 proteins in formalin-fixed and paraffin-embedded tissue specimens of OKC (n= 20) and multicystic AB (n= 20), collected from the Laboratory of Oral Pathology of the Federal University of Goiás, during the period of 2011 and 2017. Only cases with sufficient tissue and with the description of clinical characteristics were included. Symptomatic cases or cases associated with clear signs of secondary infection were not included. The clinicopathologic features of the cases evaluated are depicted in Table 1. All samples were submitted to 5-μm histologic sections and routine staining with hematoxylin and eosin, and then analyzed by an oral pathologist, under light microscopy, to confirm their histopathological diagnosis. The OKC and multicystic AB samples were taken from 40 patients with a mean age of 19.5 and 53.1 years old, respectively, and a female to male ratio of 2:1.

**Table 1:**
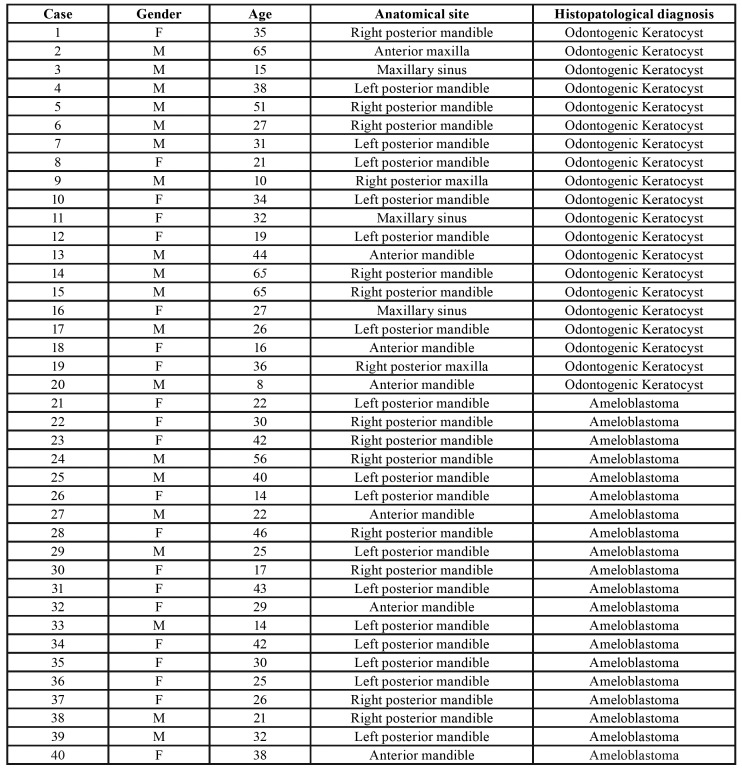
Clinical and pathological characteristics of the Odontogenic Keratocyst and Ameloblastoma.

- Immunohistochemistry

The immunohistochemical reactions for anti-SOX2 (mouse monoclonal antibody, ref: sc365823; Santa Cruz Biotechnology, Santa Cruz, CA) and anti-BCL-2 (mouse monoclonal antibody, clone 124; Dako Cytomation, Glostrup, Denmark) were performed using 3-μm 4% formalin-fixed slides submitted to dewaxing with xylene and hydration, in a series of decreasing concentrations of ethanol. Antigen retrieval was performed by immersing the sections in 10 mM monohydrated citrate buffer solution (pH 6.0), and then heating them in a microwave oven for 15 minutes. The endogenous peroxidase was consumed with 6% hydrogen peroxide and an absolute methanol solution in two baths of 15 min each at room temperature. After washing in TRIS buffer (pH 7.6), the slides were incubated with primary anti-SOX2 (dilution 1:50) and anti-BCL-2 (dilution 1:50) antibodies for 30 minutes. The slides were then exposed to an avidin–biotin complex (LSAB-Kit + HRP; Dako Cytomation, Carpinteria, CA, USA) and to 3,3′-diaminobenzidin chromogen (DAB+; Dako Cytomation, Carpinteria, CA, USA), and subsequently counterstained using Meyer’s hematoxylin, dehydrated in ethanol, cleared in xylene and cover-slipped. Sections of adenoid cystic carcinoma were used as a positive control for SOX2, whereas lymph node with follicular hyperplasia was used for BCL-2. The negative control was obtained by omitting the primary antibody during the reaction. The sections were considered positive for SOX2 when brown staining was observed in the cell nucleus, and positive for BCL-2 when staining was localized in the cytoplasmic compartment. The histological slides were digitalized under 200x magnification and the immunohistochemical expressions were analyzed using ImageJ computer software (NIH, Bethesda, MD, USA).

The SOX2 and BCL-2 immunoexpression was analyzed by a quantitative and qualitative scoring system based on the percentage of staining cells and the intensity of this staining. This scoring system designates 3 categories to evaluate the proportion of positive cells (0, no expression; 1, expression in <50% of cells; and 2, expression in >50% of cells), and another 3 categories to estimate staining intensity (0, no staining; 1, weak; and 2, strong). The percentage of positive cells and the staining intensity in the OKC epithelium were analyzed in basal and suprabasal layers separately. In AB specimens, the expression of SOX2 and BCL-2 were evaluated individually in central areas of neoplastic epithelium and in peripheral ameloblast-like columnar cells. The final score for SOX2 and BCL-2 was calculated as follows: the sum of the basal and suprabasal ratio values multiplied by the highest intensity value found in the OKC specimen. In AB, the final scores were also arrived at by adding the proportional values of the two different regions of the neoplastic epithelium (center and peripheral cells), multiplied by the highest intensity value found in the AB specimen. This system generated a score ranging from 0 to 8. The immunoexpression of the mentioned markers was evaluated at "hot spots" by two independent pathologists, blinded to all other information on the cases.

- Statistical analysis

The statistical analysis was performed with SPSS 20.0 software (Statistical Package for Social Sciences, Chicago, IL, USA). The comparison between the immunoexpression of SOX2 and BCL-2 in OKC and AB specimens was submitted to the Mann–Whitney U-test. Spearman’s correlation coefficient was performed to evaluate the correlation between SOX2 and BCL-2 expression. Statistical significance was set at *P*<0.05.

## Results

- SOX2 expression in odontogenic keratocyst and ameloblastoma

**Figure 1:**
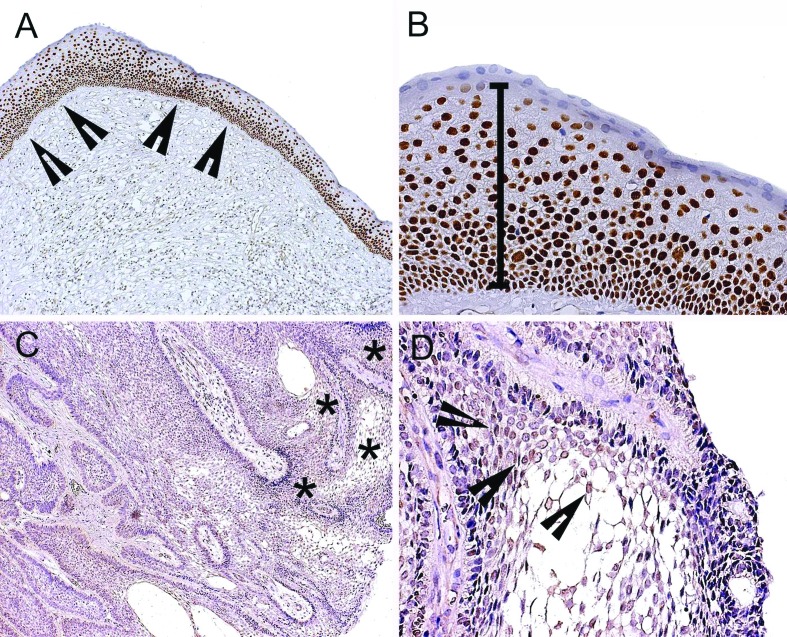
Immunohistochemical expression of SOX2 in odontogenic keratocyst (OKC) and ameloblastoma (AB) histological specimens. (A) SOX2 nuclear expression in palisade basal cell layer (arrow heads) of OKC (100x). (B) SOX2 nuclear expression in entire thickness of OKC epithelium lining (200x). (C) SOX2 shows weak nuclear expression in central areas of neoplastic epithelium nests of AB (asterisk) (100x). (D) Weak expression of SOX2 in areas resembling stellate reticulum (arrow heads), and discrete staining in peripheral ameloblast-like columnar cells of neoplastic epithelium (200x).

SOX2 immunostaining was observed in all the samples of OKC (n=20) (Table 2), with patent expression in the nucleus of basal and suprabasal cells of the epithelium lining (Fig. 1). A stronger expression of SOX2 was observed in the basal layer of OKC (Fig. 1). In multicystic AB, SOX2 expression was found in 55% (n=11) of the sample (Table 2), with a weak nuclear expression of SOX2 in the central areas of the neoplastic epithelium nests (Fig. 1), especially in areas resembling stellate reticulum (Fig. 1).

**Table 2:**
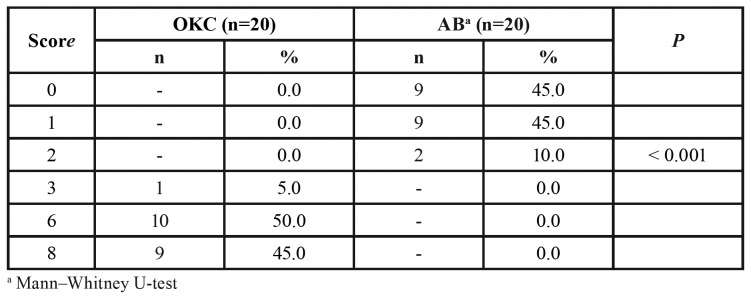
SOX2 final scores for odontogenic keratocyst (OKC) and multicystic ameloblastoma (AB).

To a lesser extent, a discrete SOX2 expression was also seen in the peripheral ameloblast-like columnar cells of the neoplastic epithelium (Fig. 1). In OKC, the final score for SOX2 ranged from 3 to 8, revealing the highest expression of this marker in OKC, in comparison with AB samples (Mann–Whitney U-test, *P*<0.05) (Table 2).

- BCL-2 expression in odontogenic keratocyst and ameloblastoma

**Figure 2:**
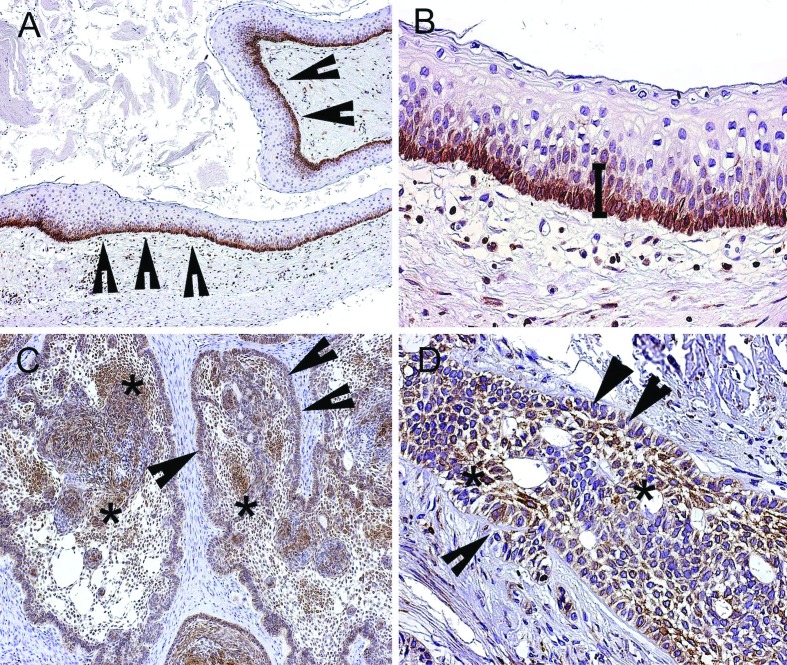
Immunoexpression of BCL-2 in the epithelium lining of odontogenic keratocyst (OKC) and ameloblastoma (AB) specimens. (A) BCL-2 staining in palisade basal cell layer (arrow heads) of OKC (100x). (B) Cytoplasmic expression of BCL-2 in basal and parabasal cell layers of OKC (200X). (C) BCL-2 staining in peripheral regions of neoplastic odontogenic epithelium nests (arrow heads) (100x). (D) Cytoplasmic expression of BCL-2 in central areas resembling stellate reticulum (asterisk) (200x).

BCL-2 expression was observed in 100% (n=20) of the OKC specimens (Table 3), showing a marked cytoplasmic BCL-2 expression in cells of the basal layer of the odontogenic epithelium lining (Fig. 2). Cytoplasmic expression of BCL-2 was also noted in 85% (n=17) of multicystic AB samples (Table 3), with an evident expression in both the central and peripheral regions of neoplastic odontogenic epithelium nests (Fig. 2). There was no significant difference in the BCL-2 immunostaining between OKC and AB, considering that their final score ranged from 1 to 6 (Table 3).

**Table 3:**
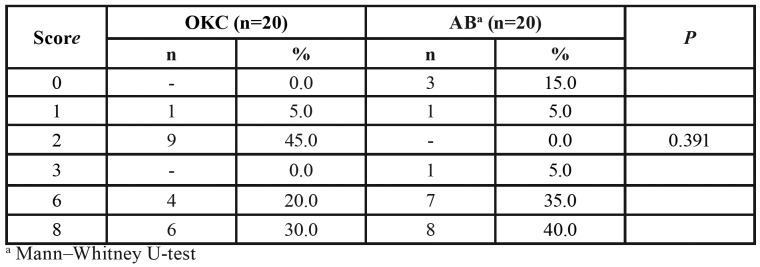
BCL-2 final scores for odontogenic keratocyst (OKC) and multicystic ameloblastoma (AB).

- Correlation of SOX2 and BCL-2 expression 

SOX2 and BCL-2 showed no significant correlation in either OKC (r = -0.5111x; *P*=0.136) or multicystic AB specimens (r = -0.7018x; *P*= 0.461).

## Discussion

Previous WHO classification of head and neck tumors have aroused extensive discussion around the nature of OKCs (3). In 2005, the WHO consensus group reclassified OKC as a benign odontogenic tumor derived from the odontogenic epithelium. This classification was based on the growth potential, the putatively high recurrence rate, and the presence of mutations in OKC specimens (3). However, since these lesions could regress following decompression procedures, and could also present molecular alterations that are more frequently observed in syndromic cases (nevoid basal cell carcinoma syndrome), the WHO 2017 expert panel recognized OKC as a developmental odontogenic cyst (23). It is believed that the lack of evidence supporting the neoplastic nature of OKC was taken into account in the last WHO classification, and the assumption that this lesion actually does not constitute a tumor was discussed (23). These issues reflect the ongoing studies on OKC molecular characteristics, which have focused on expounding the reasons for its locally aggressive biological behavior and its continuous growth potential.

SOX2 and BCL-2 have a significant role in oncogenic signaling pathways, involved in cell proliferation and apoptosis (24), and their expression can be found in OKC (9,18). In the present study, we aimed to analyze the immunohistochemical expression of SOX2 and BCL-2 in OKC, and to identify a possible correlation in their expression. Additionally, since AB is a well-recognized, locally aggressive and recurrent odontogenic tumor, we also sought to compare its expression with the OKC expression. In this case, we found a greater expression of SOX2 in OKC than AB, with noticeable staining in all cell layers of the cyst lining. This high expression of SOX2 could denote that OKC cells have significant self-renewal and proliferative properties (16,25). This would suggest that there is an imbalance between cell growth and cell death in OKC, which could be a sign of neoplastic behavior (26). In addition, SOX2 expression could also imply that a particular group of cells has stem cell properties, contributing to maintain clonogenicity (27). The weak or absent expression of SOX2 in AB specimens found in the present study could imply that this particular odontogenic tumor presents another molecular pathway responsible for conserving its neoplastic behavior.

SOX2 overexpression is observed in different types of malignancies (28), including squamous cell lung carcinomas, squamous cell esophageal carcinomas (29), oral squamous cell carcinomas (30), melanoma, sinonasal carcinomas, Ewing’s sarcoma (28) and ameloblastic carcinoma (31). Despite the marked expression of SOX2 in cancer, this protein is also expressed in the epithelium of developing teeth (16), in the surface epithelium of oral mucosa and in the epithelium lining of dentigerous cysts (31).

In the present study, SOX2 staining was noted in the entire thickness of the OKC epithelium lining, which could imply that all cell layers of OKC specimens preserve progenitor characteristics of odontogenic epithelium, presenting a stem cell-like phenotype, and a continuous growth (32). This expression, in a very cautious conjecture, could be interpreted as part of the physiological expression of SOX2 related to epithelium turnover, restricted to cells of the stratified epithelium proliferation zone, more specifically those in the basal cell layer (33). However, in the present study, SOX2 staining was also found in the intermediate and surface cells, which potentially indicates that the upper layer cells of the OKC epithelium lining also present a proliferative potential and stem cell properties (16,25).

Regarding this proliferative potential, the findings of a previous investigation with specific markers are consistent with ours, namely the higher proliferative activity in the intermediate layers of OKC (33). According to Kichi *et al*. (33) [2005], the Ki-67-positive ratio observed in the OKC epithelium lining was about 15% in the basal layer and close to 36% in the intermediate layer. Their results, in association with the SOX2 expression observed in the present investigation, indicate that the intermediate cell layer of OKC possesses significant proliferative activity.

Understanding the pathogenesis and nature of OKC is important in order to clarify its aggressive nature, and also propose new treatment modalities (4). There are many molecular alterations related to sporadic and syndromic OKC cases, several of which could influence important cell signaling pathways (4,34). One of the main genetic changes described in OKC is the dysregulation of the hedgehog (Hh) signaling pathway caused by mutations in the Patched transmembrane spanning receptor 1 (PTCH1) (4). Hh is a central controller in embryonic development organization, participating in cell proliferation, cell fate determination, differentiation and stem cell maintenance (35). Hh hyperactivation is responsible for neoplastic transformation in different types of tumors (35), according to several modes of pathway activation described in the literature (36). One of the main Hh pathway activators is characterized by the binding of Hh to PTCH1 and consequent release of the smoothened (SMO) of the repressive action of PTCH1 (36). SMO thus participates in activating Gli, which mediates the Hh target gene expression (13). In fact, Hh activity may include the expression of genes that affect proliferation, cell survival and angiogenesis (37).

The Hh downstream protein Gli1 can regulate BCL-2 (37), an important anti-apoptotic gene, which encodes a protein capable of preventing apoptosis by enabling cell survival independently of cell division (38). In this study, we found a significant BCL-2 expression in OKC and AB. It was observed that the BCL-2 staining in OKC specimens was restricted to the cytoplasm of cells of the lower third of the epithelium lining. Previous investigations have also shown that BCL-2 expression is increased in OKC in the basal layer cells (18). This BCL-2 expression could contribute to inhibit apoptosis in OKC cells, by allowing genetically unsTable cells to survive and accumulate additional mutations that eventually lead to its neoplastic transformation (39). However, the single expression of BCL-2 is not enough to endorse the neoplastic nature of OKC, although it has been found to be significantly expressed in AB, a lesion with a recognized neoplastic nature.

Here we found that OKC expresses SOX2 and BCL-2 proteins in a significant manner, but with no statistical correlation between them. Nevertheless, it is plausible to assume that their expression in OKC is not just a coincidence, since both are related to the Hh signaling pathway (35,39,40) and both seem to be influenced by miR-15a (24,41). Our findings are in sync with those of other studies that also found SOX2 and BCL-2 expression in OKC (9,18). However, none of these investigations proposed to analyze the expression of these two markers together, or even made any consideration regarding their relationship. As mentioned before, Hh signaling pathway dysregulation is involved in anti-apoptotic ability, enhanced proliferative capacity and maintaining the stemness of tumor cells (35). There is evidence that the anti-apoptotic action of BCL-2 is a downstream target of the Hh pathway (35), and acts to promote tumor cell survival. In addition, there is evidence that SOX2 is an upstream protein of the Hh signaling pathway, and acts to alter the cell proliferation potential and maintain the stemness of tumor cells (39). Diniz *et al*. (41) [2012] demonstrated the epigenetic mechanism (miRNAs) that might lead to the regulation of BCL-2 expression in OKC. According to their study, miR-15a overexpression seems to downregulate BCL-2, acting as a suppressor of this apoptotic gene. Curiously, this influence of miR-15a in BCL-2 levels was also demonstrated in colon cancer (24). In the study mentioned, the overexpression of miR-15a showed downstream suppression of BCL-2 and SOX2 proteins (24).

The significant expression of SOX2 and BCL-2 observed in the present investigation with OKC specimens could offer a hint into their biological nature and pathogenesis. Although the immunohistochemical expression of only two markers is insufficient to prove that OKC is a cystic tumor, our results suggest that SOX2 and BCL-2 are related to the persistent growth potential of OKC, a characteristic compatible with its neoplastic nature. In summary, a higher expression of SOX2 was observed in OKC specimens in comparison with AB. BCL-2 was significantly expressed in OKC and AB. Although there was no statistical association between SOX2 and BCL-2, we believe that their expression could be related to the proliferative and anti-apoptotic capability of the OKC epithelium lining. Therefore, we suggest that SOX2 and BCL-2 are somehow associated with the biological behavior of OKC, and that the higher expression of SOX2 found in the present study might be an upstream influence on the Hh signaling pathway.
